# Supplementary material for: Wafer-scale nanofabrication of telecom single-photon emitters in silicon
Source: Nat Commun. 2022 Dec 12;13:7683. doi: 10.1038/s41467-022-35051-5 (PMC9744816; doi:10.1038/s41467-022-35051-5)
Supplement: Supplementary file 1 — Supplementary Information [file 41467_2022_35051_MOESM1_ESM.pdf]

Supplementary Information

Wafer-scale nanofabrication of telecom single-photon emitters in silicon

M. Hollenbach[1,2], N. Klingner[1], N. S. Jagtap[1,2], L. Bischoff[1], C. Fowley[1], U. Kentsch[1], G. Hlawacek[1], A. Erbe[1], N. V. Abrosimov[3], M. Helm[1,2], Y. Berencén[1], and G. V. Astakhov[1]

[1] *Helmholtz-Zentrum Dresden-Rossendorf, Institute of Ion Beam Physics and Materials Research, 01328 Dresden, Germany*

[2] *Technische Universität Dresden, 01062 Dresden, Germany*

[3] *Leibniz-Institut für Kristallzüchtung (IKZ), 12489 Berlin, Germany*

Contents

Experimental setup 2

Creation statistics of single G and W centers 3

Supporting tables 5

Supporting figures 7

EXPERIMENTAL SETUP

The experimental setup for single-defect spectroscopy is schematically shown in Supplementary Fig. 2. The confocal imaging system is built-up based on a customized closed-cycle helium cryostat (attocube, attoDRY800) that ensures a stable sample temperature of 6.3 K. The laser light is directed through fiber optics and reflected by a dichroic beam-splitter (DCM1) into a high NA cryo-compatible microscope objective (attocube, LT APO IR, NA = 0.81), resulting in a diffraction-limited focal spot of 1 μm and a uniform excitation field on the silicon sample plane. For two-dimensional confocal intensity maps, photostability, laser power saturation measurements, and PL spectra, a continuous wave (cw) single-mode 637-nm laser diode (Thorlabs, LP637-SF70) is used. For PL decay measurements, the 785-nm laser light (Thorlabs, LP785-SF20) is pulsed using a fiber-coupled acousto-optical modulator (AA optoelectronic, MT250-NIR6) with a rise time of nominally 6 ns. For both cw and pulsed excitation, the laser power is controlled using in-line variable fiber optical attenuators (Thorlabs, VOA630-FC/VOA780-FC). To prevent residual BG contribution from the laser lines, the excitation path is cleaned-up with an 800-nm shortpass filter (SP) (Thorlabs, FESH0800). Where quoted, the laser power is measured before the objective and consequently represents an upper limit.

Using three linear nanopositioners (attocube, ANPx311) for precise and independent motion control in the lateral and axial direction (6 mm range and 200 nm precision) anchored to the sample holder, three-dimensional confocal PL raster scans are performed. The PL from emitting color centers is collected by the same objective and reflected into the detection path by a second dichroic beamsplitter (DCM2) before being imaged onto a 1 mm confocal pinhole (spatial filter).

A set of 1200 nm longpass filters (LP) (Thorlabs, FELH1200) are used to fully remove the residuals of the laser light and unwanted Si bandgap contribution. Depending on the type of the emitter under study, we use a bandpass (BP) centered either at 1225 nm (Edmund Optics, 87-864) or 1275 nm (Edmund Optics, 87-865), each with a transmission window of 50 nm. To spectroscopically separate the superimposed background PL from the emission of the G center in the non-annealed wafer, we use a narrow bandpass (BP) filter with a central wavelength of 1278 nm and a nominal bandwidth of 1 nm, matching the ZPL position of the G center.

The emitted photons are detected by a broadband fiber-coupled superconducting nanowire single-photon detector (SNSPD) module (Single Quantum Eos) with a time jitter not exceeding 40 ps. To measure PL spectra, the signal is redirected into an IR spectrometer equipped with a Peltier-cooled InGaAs photodetector array (PDA)(Andor Technology, iDus 1.7 μm). The SNSPD is used for the spectrally integrated PL measurements and features a detection efficiency of $> 90\%$ at 1.3 μm, whereas the PDA provides a quantum efficiency of around 80% in the wavelength range from 1.0 μm to 1.6 μm. To prove the single-photon nature, we use two SNSPDs in Hanbury Brown and Twiss (HBT) configuration. To this end, we split the PL signal into two detection arms using a 50/50 fiber-optic wideband beamsplitter (Thorlabs, TW1300R5F1).

The lifetime measurements and the photon statistics are recorded with a time-to-digital converter (Swabian Instruments, Time Tagger 20) with a time jitter of 14 ps. The synchronization of the measurements is realized by a digital pattern and arbitrary waveform generator (Swabian Instruments, Pulse Streamer 8/2).

CREATION STATISTICS OF SINGLE G AND W CENTERS

The creation of optically-active point defects or color centers in solids by ion implantation is a stochastic process. In case of single-atom defects, such as the silicon vacancy in SiC or the boron vacancy in hBN, a successful implantation of even a single ion can result in the formation of such color centers, though not every implantation event is successful. The creation yield – the inverse of the average number of ions required to create a single color center ($\gamma = 1/\overline{n}$) – is usually much below $\gamma < 100\%$ despite ion cascades during implantation. This is because the implantation should create the color center (for instance, vacancy) in a

certain charge state, minimally destroy the lattice structure in its vicinity and it should be spatially isolated from other damage-related defects. The statistical distribution of single-atom color centers can then be well described by the Poisson function $P_\mu(m) = \mu^m e^{-\mu}/m!$. Here, m is the number of color centers created during the implantation and μ is the expected value. If the implantation is optimized for the creation of single color centers $\mu = 1$ using $\overline{n}$ ions, the probability is $P_1(0) = P_1(1) = 36.8\%$. This is also the maximum probability for the Poisson distribution among all possible μ and m.

Our analysis presented in Fig. 4e of the main text shows a probability of $(56 \pm 28)\%$ for the creation of single W centers. Though within the error bars it falls below the maximum value of 36.8%, there is a strong experimental indication for sub-Poisson distribution of the W centers. We expect this behavior for composite defects, i.e., consisting of more than one atom. For a qualitative explanation, we consider the Poisson distribution of successfully implanted Si ions with $\mu = 4$ (Supplementary Fig. 4a). Under "successful implantation" we understand here the implantation of one over three Si atoms in the tri-interstitial complex forming the W center. Under this assumption, the creation of single and two W centers requires 3 and 6 successful Si implantations, correspondingly. The summation gives probabilities of 23.8%, 54.7%, 19.5% and 2.0% for zero, single, two and three W centers, respectively (Supplementary Fig. 4b).

Similar arguments can be applied for the formation of the G center, consisting of two C atoms and one Si atom. Because the probability to find two C atoms in the neighboring crystal sites is vanishingly small, the formation of a single G center can be considered as a multi-step implantation process: (1) implanted Si ion kicks out a Si atom from a lattice site adjacent to a C substitutional and forms a vacancy, (2) implanted Si ion kicks out a C atom from one substitutional into the vacancy and forms a C pair, (3) implanted Si ion creates silicon interstitial adjacent to the C substitutional pair and forms a G center. The real formation processes of the G (and W) centers under Si implantation are much more complex than in our simplified consideration and beyond the scope of this work. Nevertheless, our approach for the calculation of sub-Poisson distribution reproduces well experiments in Fig. 2b and Fig. 4e with $\mu = 4$ and $\mu = 4.8$, respectively.

SUPPORTING TABLES

TABLE 1: Average number of implanted Si ions per spot in the FIB pattern.

Row	Average Si ions per spot
1	6
2	9
3	13
4	16
5	25
6	33
7	45
8	61
9	83
10	113
11	153
12	208
13	283
14	384
15	570

TABLE 2: Variation of the nanohole diameter in the PMMA pattern for the creation of G amd W centers.

Row	Nominal hole diameter (nm) G center	Nominal hole diameter (nm) W center
1	30	50
2	35	60
3	40	70
4	45	80
5	50	90
6	55	100
7	60	110
8	65	120
9	70	130
10	75	140
11	80	150
12	85	200
13	90	300
14	95	400
15	100	500
16	125	600
17	150	700
18	200	800
19	300	900
20	400	1000
21	2000	2000

SUPPORTING FIGURES

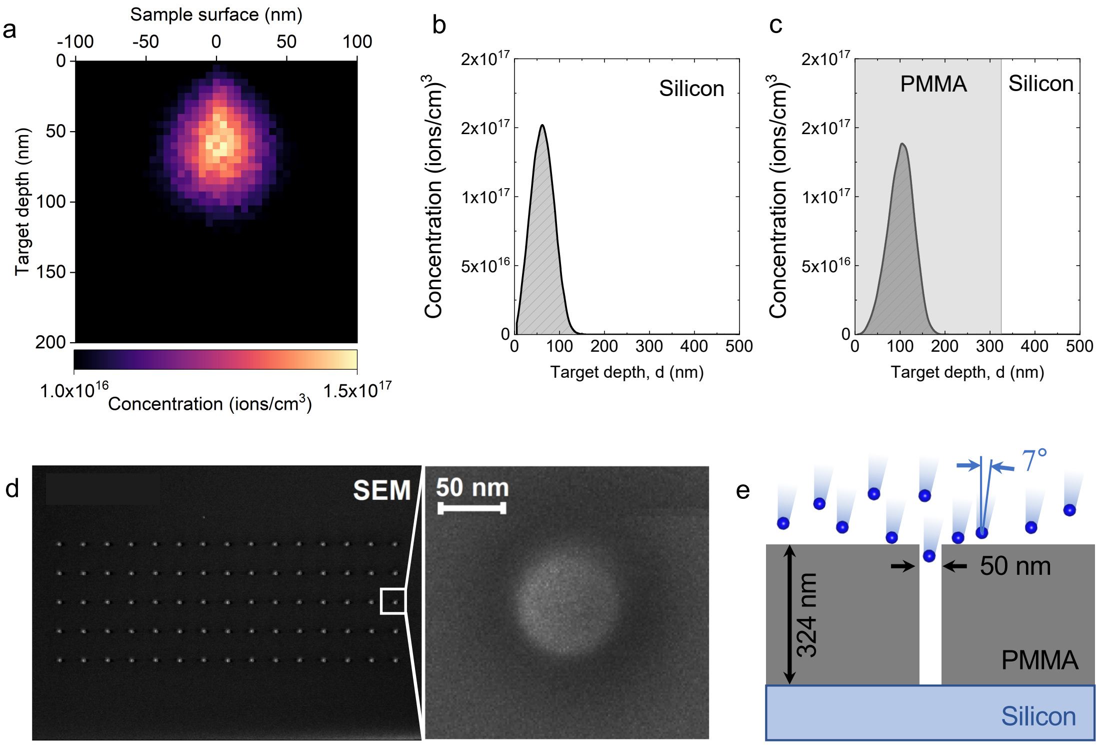

FIG. 1: a, Spatially resolved SRIM simulation of the implantation with double charged Si^{2+} ions with a landing energy of 40 keV into the Si lattice indicating longitudinal and lateral straggling. b, SRIM simulation of the Si implantation stopping range in Si lattice ($R_p \sim 60$ nm). c, SRIM simulation of the Si implantation stopping range in PMMA ($R_p \sim 100$ nm). d, SEM image of a sample subset of nanohole arrays patterned on the PMMA surface. Zoom-in shows an SEM micrograph of an aperture with a nominal diameter of approximately $d = 50$ nm. e, Schematic cross-section of the Si implantation through a nanohole in the PMMA mask with a nominal thickness $t = 324$ nm. Implantation is performed at $\theta = 7°$ tilt to avoid ion channeling.

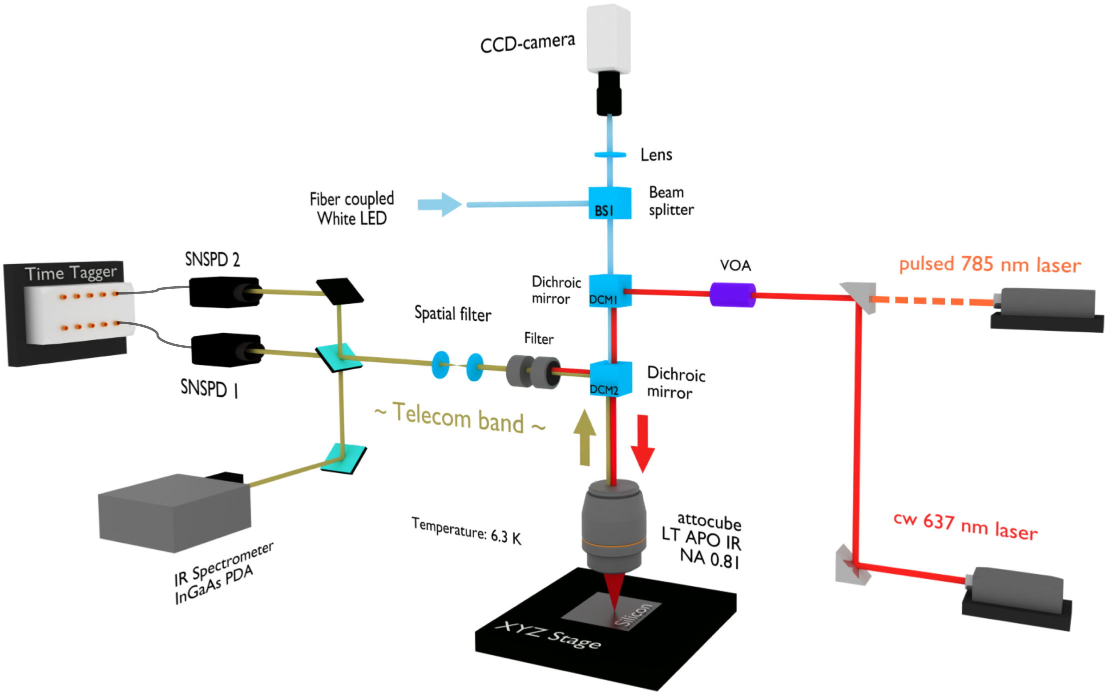

FIG. 2: A scheme of the experimental setup. The PL is excited with either cw 637-nm or pulsed 785-nm laser. The PL is collected through optical LP and BP filters followed by a spatial filter. The detection is performed either with two SNSPDs or an InGaAs PDA.

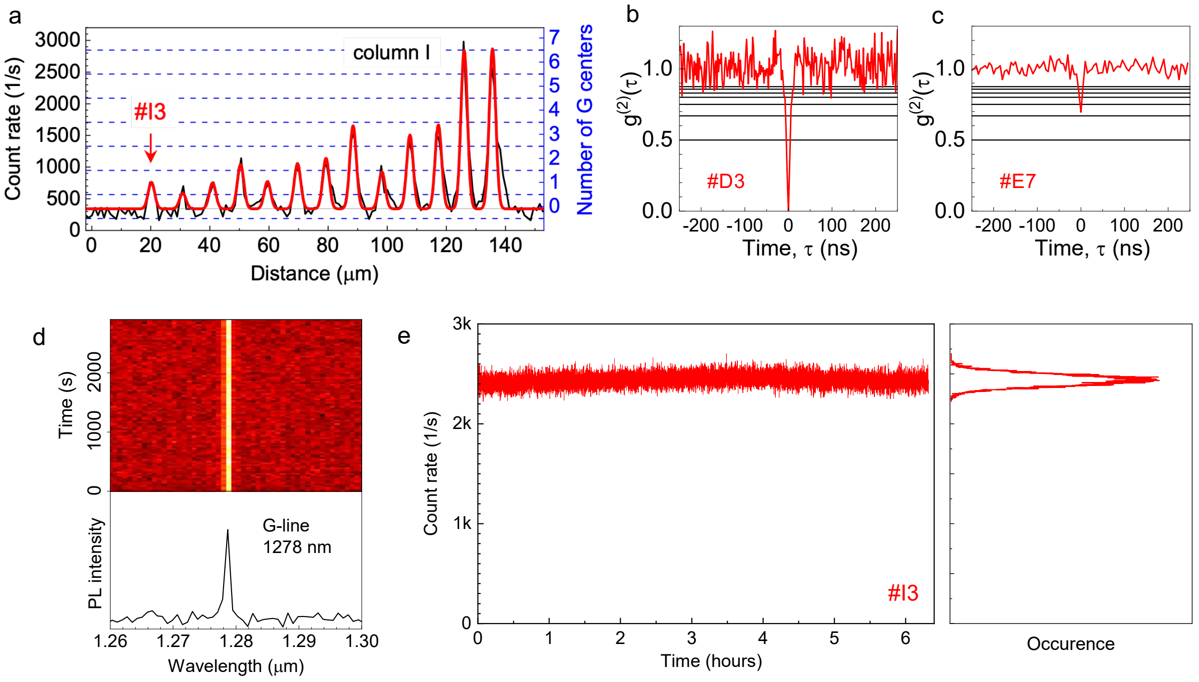

FIG. 3: a, G center ZPL line scan through column I of the FIB pattern in Fig. 1 of the main text. The red line is a multi-Gauss fit. The right axis represents the number of G centers (N) according to the count rate. b, Second-order autocorrelation function $g^{(2)}(\tau)$ obtained at spot #D3 with BG correction, yielding $N = 1$. c, Second-order correlation function $g^{(2)}(\tau)$ obtained at spot #E7 with BG correction, yielding $N = 3$. d, ZPL time stability of a single G center obtained with a 1-nm BP filter about one hour. e, PL time trace of a single G center without BP filter for over 6 hours.

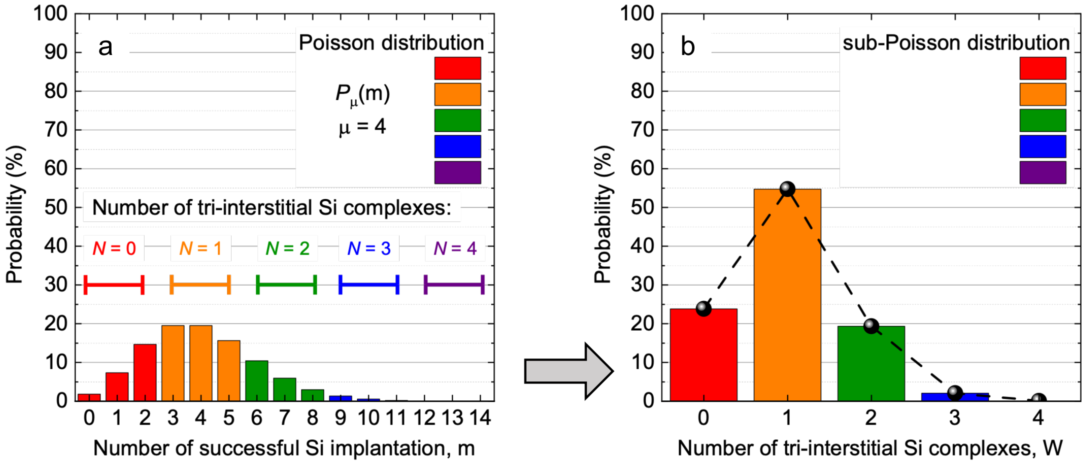

FIG. 4: a, Poisson distribution $P_\mu(m)$ of successfully implanted Si ions with expected value $\mu = 4$ per each implantation. b, Corresponding distribution of tri-interstitial Si complexes with sub-Poisson statistics.

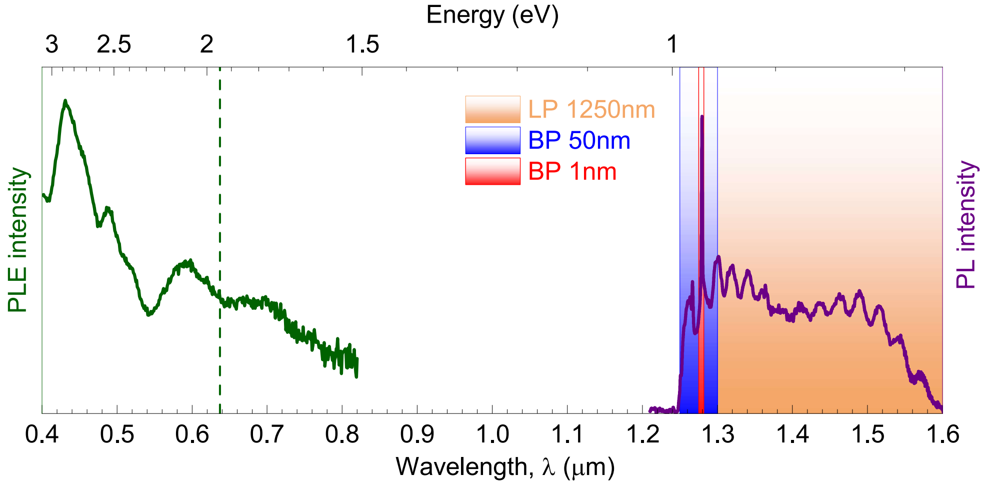

FIG. 5: PL spectrum of a single G center before annealing and PLE spectrum of the G center ensemble created by broad-beam implantation. The PLE intenisty is monitored at the ZPL position $\lambda_G = 1278\,\mathrm{nm}$. The vertical dashed line indicates the excitation wavelength $\lambda_{exc} = 637\,\mathrm{nm}$.

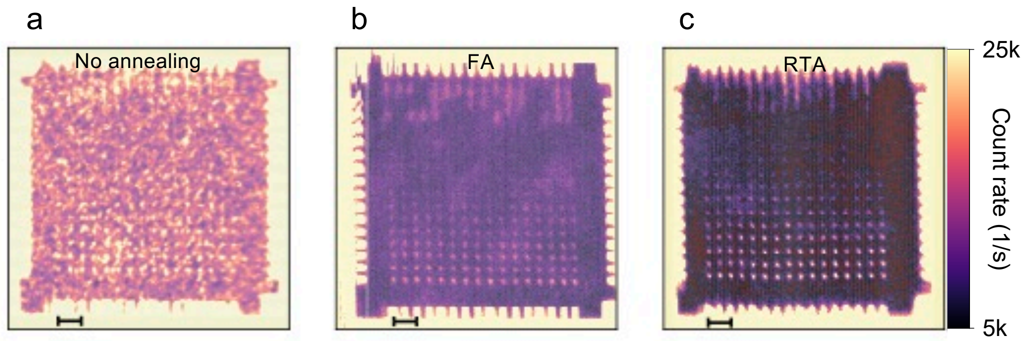

FIG. 6: a, Confocal PL intensity map as in Fig. 1 of the main text but measured with a long-pass filter $\lambda > 1250\,\mathrm{nm}$ without annealing. b, Confocal PL intensity map after furnace annealing (FA) at 500°C over 2 hours in an N_2 atmosphere. The FIB pattern is created after annealing. c, Confocal PL intensity map after rapid thermal annealing (RTA) at 1000°C over 60 seconds in an N_2 atmosphere. The FIB pattern is created after annealing. The scale bar in a-c is 20 μm.

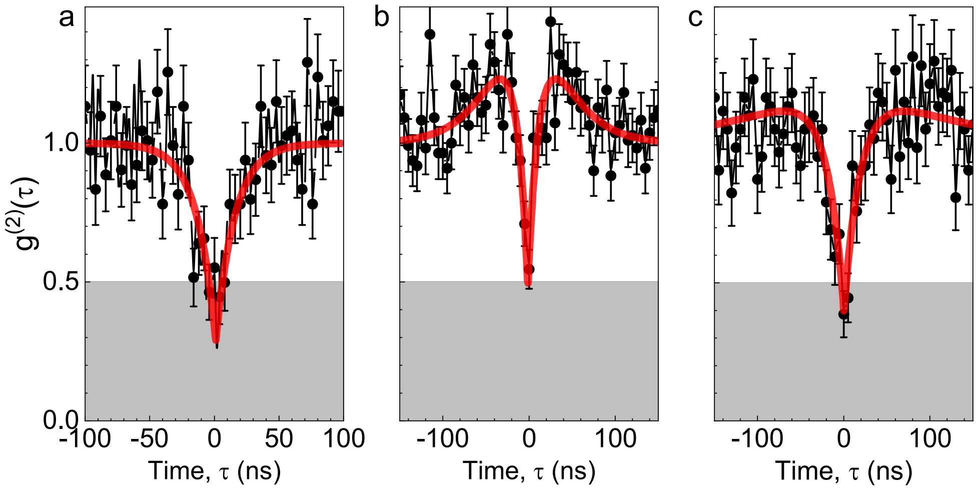

FIG. 7: Examples of the second-order autocorrelation functions $g^{(2)}(\tau)$ obtained in an annealed SOI wafer after broad-beam Si implantation through PMMA nanoholes (Fig. 2 of the main text). No BG corrections are performed. In all cases, the number of G centers is $N = 1$.

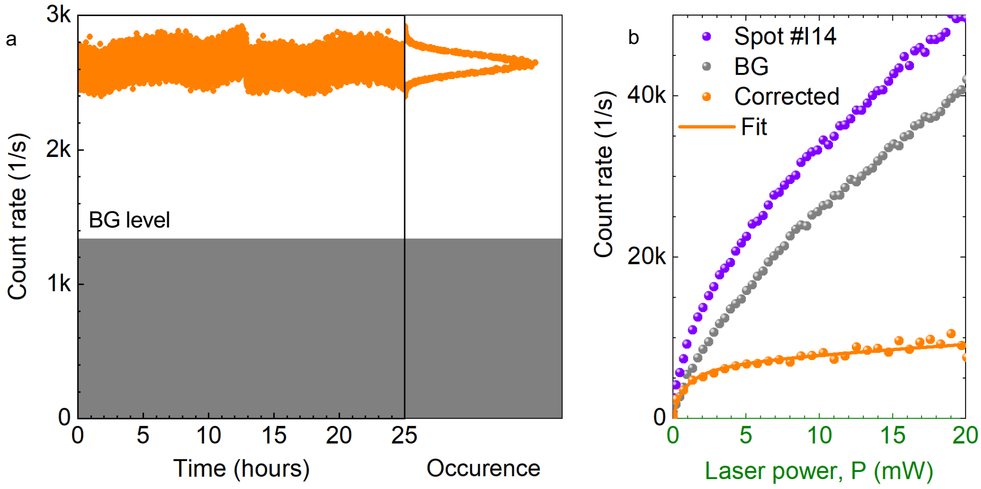

FIG. 8: a, PL time trace of a single W center (spot #I14) with a BP filter $\Delta\lambda = 50\,\text{nm}$. b, Count rate of a single W center as a function of the excitation power in the presence of BG. The power dependence of the BG is also shown for comparison. The corrected power dependence corresponds to Fig. 4 of the main text.

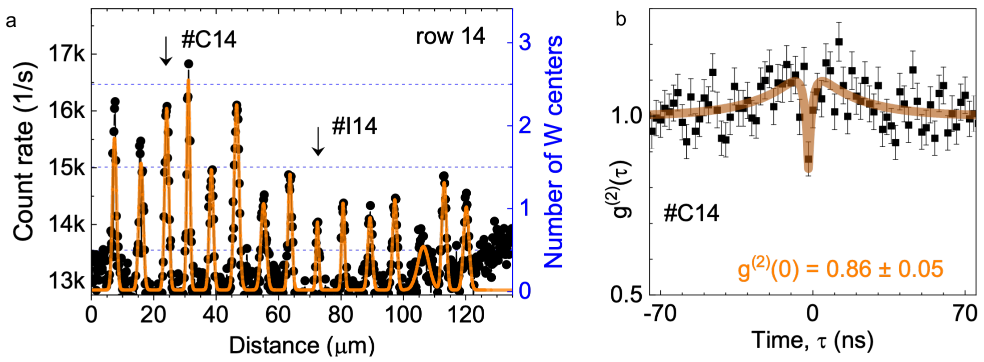

FIG. 9: a, W center PL line scan through the line 14 of the FIB pattern in Fig. 4 of the main text. The orange line is a multi-Gauss fit. The right axis represents the number of W centers (N) assuming the count rate from each spot to be proportional to the number of W-centers within this spot. b, Second-order autocorrelation function $g^{(2)}(\tau)$ obtained at spot #C14 without any BG correction. The BG correction gives $g^{(2)}_{corr}(0) = 0.52 \pm 0.15$, corresponding two $N = 2$.
